# Solid component tumor doubling time is a prognostic factor in non-small cell lung cancer patients

**DOI:** 10.1186/s13019-019-0879-x

**Published:** 2019-03-12

**Authors:** Kentaro Miura, Kazutoshi Hamanaka, Tomonobu Koizumi, Satoshi Kawakami, Nobutaka Kobayashi, Ken-ichi Ito

**Affiliations:** 1 Department of Breast, Endocrine and Thoracic Surgery, Shinshu University, 3-1-1 Asahi, Matsumoto, 390-8621 Japan; 2 Department of Comprehensive Cancer Therapy, Shinshu University, Matsumoto, Japan; 3 Department of Radiology, Shinshu University, Matsumoto, Japan; 4 Department of Thoracic Suegery, Japanese Red Cross Society Nagano Hospital, Nagano, Japan

**Keywords:** Lung cancer, Tumor doubling time, Solid component, Prognosis

## Abstract

**Background:**

Recently, several reports investigating tumor doubling times (TDTs) in lung cancer have demonstrated that lung cancer patients with shorter TDTs have poor prognoses. Although data have shown that the solid component of a tumor is clinically more important, relationships between solid component TDTs and lung cancer prognoses remain unclear.

**Methods:**

To evaluate relationships between TDT and survival, we retrospectively evaluated 231 patients who underwent surgical resection for non-small cell lung cancer. The TDTs of whole and solid components were calculated using preoperative thin-slice chest computed tomography scans with a cut-off of 400 d between scans.

**Results:**

Patients with short TDTs (< 400 d) both in the solid and whole components had poor prognoses. Among pathological stage I patients (*n* = 176), short solid component TDT (< 400 d) significantly influenced prognosis only in pathological stage IB patients. Moreover, we found that patients with shorter solid component TDTs had significantly worse prognosis compared with patients who showed shorter whole component TDTs.

**Conclusions:**

Short solid component TDTs (< 400 d) could be a poor prognostic indicator for non-small cell lung cancer patients undergoing surgical resection; furthermore, clinicians should pay particularly close attention to cases with rapid growth of the solid tumor component.

## Background

Worldwide, lung cancer is one of the most lethal malignant tumors. Recently, there have been several reports focused on the tumor doubling time (TDT) of lung cancer [1–7]. These studies have found relationships between TDT and lung cancer prognosis using chest radiograph and computed tomography (CT) scans: longer TDT is associated with better prognosis. Some reports have suggested that the optimal cut-off for TDT is 400 d, as this can distinguish between indolent and malignant lesions [4, 5, 8]. Furthermore, the nodule management strategy of the Dutch–Belgian lung cancer screening trial (NELSON) demonstrated the high sensitivity and specificity of lung cancer detection using TDT [4]. Thus, the use of TDT as a prognostic indicator for lung cancer has become more common.

The 8th edition of the American Joint Committee for Cancer Staging System for lung cancer was revised to focus on the diameter of the solid component in a chest CT scan. Particularly, stage I disease was subdivided into stages IA1, IA2, IA3 and IB depending on the size of solid components in chest CT scans. To our knowledge, there have been no reports regarding relationships between solid component TDT and prognosis in surgically resected non-small cell lung cancer (NSCLC) patients; furthermore, there are no reports of using TDT as a prognostic evaluation for pathological stages IA1, IA2, IA3 and IB according to the 8th edition of the American Joint Committee for Cancer Staging System.

This study was performed to determine whether solid component TDT is related to prognosis in NSCLC patients undergoing surgical resection compared with TDT of all components, and to clarify the relationships between TDT of solid components in pathological stage IA1, IA2, IA3 and IB NSCLC patients. These results could influence postoperative therapeutic options or lengths of observation periods.

## Methods

### Patient population and study design

**Fig. 1:**
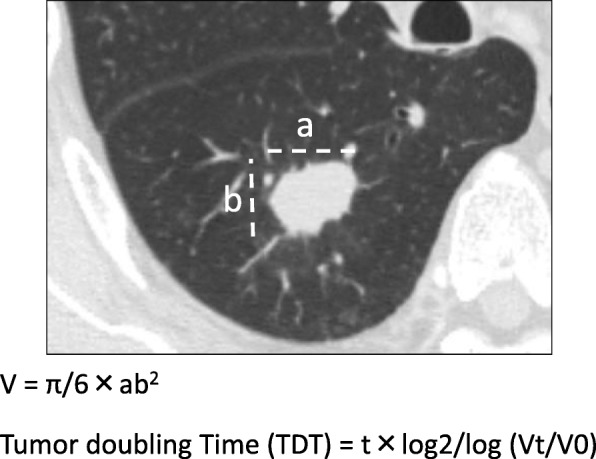
CT findings and the Schwartz equation used to determine tumor volumes. a, maximum tumor diameter; b, largest perpendicular tumor diameter; t, time between the two CT scans; Vt, tumor volume from preoperative CT scan; V0, tumor volume from initial CT scan

We indicate the patient selection and exclusion. We evaluated 717 NSCLC patients who were treated with curative surgical resection between January 2006 and December 2012 at our institute. Among them, 486 patients were excluded because of wedge resection (*n* = 149; systematic lymph node dissection was not performed), preoperative chemotherapy and/or radiotherapy (*n* = 10), no preoperative CT scans (at least two) within the last 2 months (n = 149), pathological stage 0 disease (*n* = 38), and tumors whose size could not be evaluated because of inflammatory changes or obstructive pneumonia surrounding the tumor or lung cavity (*n* = 151). The remaining 231 patients were reviewed, and TDTs of solid components and whole tumors were calculated from chest CT scans as shown in Fig. 1. Among the included patients, 138 had TDT < 400 d, while 93 (including the patients with infinite TDT) had TDT ≥400 d. Using clinicopathological data collected at intake, we investigated patient characteristics including age, sex, smoking history, serum carcinoembryonic antigen (CEA), pathological stage and histological subtype. We used the 8th edition of the American Joint Committee for Cancer Staging System to evaluate tumor, node, and metastases staging [9]. This study was approved by our institution’s research ethics committee (No. 3395).

### CT protocol and TDT calculations

All CT scans were performed by thin-slice CT (1.25-mm-thick sections) at our institute. The CT models used were Light Speed Ultra and Light Speed 16 (GE Healthcare, Tokyo, Japan [2006]), Light Speed VCT (GE Healthcare [between 2008 and 2012]), and Discovery CT 750 HD (GE Healthcare [after November 2011]). All tumors were measured using the caliper tool of the software program in the lung field window with the settings (WL: − 700 HU and WW: 1500 HU). Whole tumor diameters, including the ground-glass opacity (GGO) component and the solid component, were measured by two thoracic surgeons blinded to lung field condition. The average of the measurements by the two thoracic surgeons was used. If their data differed by over 2 mm, the images were reviewed and measured again. Then volume doubling times were calculated using the Schwartz formula [10, 11], which was also used in a previous study by Aoki et al. [3]:

1 $$ \mathrm{Tumor}\ \mathrm{volume}\ \left(\mathrm{V}\right)=\uppi /6\times {\mathrm{ab}}^2 $$

2 $$ \mathrm{Tumor}\ \mathrm{doubling}\ \mathrm{time}\ \left(\mathrm{TDT}\right)=\left(\mathrm{t}\times \mathrm{log}2\right)/\log\ \left(\mathrm{Vt}/\mathrm{V}0\right) $$

where, a = maximum tumor diameter; b = largest perpendicular tumor diameter; t = time between the two measurements; Vt = tumor volume from the most recent scan; and V0 = tumor volume from the initial scan.

The TDT cut-off value was set at 400 d according to previous studies [4, 5, 8], where TDT < 400 d indicated rapid growth and TDT ≥400 d indicated slow growth. If TDT was calculated to be infinity, it meant the size had not changed for over 2 months; these patients were grouped into the TDT ≥400 d group.

### Follow-up

Almost all patients visited the hospital at least every 3 months during the 5 years post-surgery, and tumor markers including CEA were evaluated and chest X-ray examinations were performed. Chest CT scans and brain magnetic resonance imaging (MRI) were performed at least once per year. Almost all patients were completely followed-up up to August 2016. Overall survival (OS) was defined as the time from surgery to death or the date of latest follow-up, and recurrence-free survival (RFS) was defined as the time from surgery to recurrence or non-lung cancer-related death.

### Statistical analysis

Continuous variables were analyzed by Student’s *t* test, and categorical variables were analyzed by Chi square test or Fisher’s exact test. OS and RFS curves were drawn using the Kaplan–Meier survival method and compared using the log-rank test. Univariate and multivariate analyses were performed using Cox proportional hazard regression models. All statistical analyses were performed using SPSS version 32 (IBM, Armonk, NY, USA) with *p* < 0.05 indicating statistical significance.

## Results

### Clinical characteristics of all patients and patients divided by TDT

We measured maximum tumor diameters and the largest perpendicular tumor diameters of whole tumors and the solid components to subsequently calculate TDTs for solid components and whole tumors using the Schwartz formula (Fig. 1). Table 1 shows the clinical characteristics of all patients. The TDT of all components ranged from 19.14 d to 30,417.42 d (median: 230.4 d), and the number of infinite TDTs was 24. For comparison, the TDT of solid components ranged from 10.73 d to 15,393.04 d (median: 175.5 d), and the number of infinite TDTs was 30.

**Table 1:** Patient characteristics according to TDT (< 400 and ≥ 400) (*n* = 231)

	All patients	TDT < 400	TDT ≥ 400	*p-value*
	*n* = 231	*n* = 138	*n* = 93	
Age (mean ± SD)	69.7 ± 9.4	70.8 ± 9.2	68.2 ± 9.5	0.046
Sex (Male/Female)	146 (63.2%)/85 (37.8%)	100 (72.5%)/38 (27.5%)	46 (49.5%)/47 (50.5%)	< 0.001
Smoking History (Ever/Never)	138 (59.7%)/93 (40.3%)	98 (71%)/40 (29%)	40 (43%)/53 (57%)	< 0.001
Serum CEA (mean ± SD)	4.53 ± 2.7	4.7 ± 8.8	4.2 ± 8.7	0.624
Type of resection				
Segmentectomy	24 (10.4%)	12 (8.7%)	12 (12.9%)	0.428
Lobectomy	206 (89.2%)	125 (90.6%)	81 (87.1%)	
Pneumonectomy	1 (0.4%)	1 (0.7%)	0	
Pathological Stage				
IA1	49 (21.2%)	17 (12.3%)	32 (34.4%)	
IA2	61 (26.4%)	34 (26.4%)	27 (29%)	
IA3	20 (8.7%)	13 (9.4%)	7 (7.5%)	
IB	46 (19.9%)	31 (22.5%)	15 (16.1%)	
IIA	26 (11.2%)	21 (15.2%)	5 (5.4%)	
IIB	16 (6.9%)	12 (8.7%)	4 (4.3%)	
IIIA	13 (5.6%)	10 (7.2%)	3 (3.2%)	
Histological type				
Adenocarcinoma	163 (70.6%)	78 (56.5%)	84 (90.3%)	< 0.001
Squamous cell carcinoma	44 (19%)	38 (27.5%)	6 (6.5%)	
Others	25 (10.8%)	22 (15.9%)	3 (3.2%)	
Solid component				
Pure solid/Mixed GGO	114 (49.4%)/117 (50.6%)	80 (57.8%)/58 (42.2%)	34 (36.6%)/59 (63.4%)	< 0.001
Tumor doubling time (all component)				
Infinite	24 (10.4%)			
range (median, excluding infinite)	19.14–30,417.42 (230.4)			
Tumor doubling time (solid component)				
Infinite	30 (13%)			
range (median, excluding infinite)	10.73–15,393.04 (175.5)			

*TDT* Tumor Doubling Time, *SD* Standard deviation

Additionally, Table 1 shows the clinical characteristics of the NSCLC cases divided by TDT at 400 d. The mean age was higher in the TDT < 400 d group than in the TDT ≥400 d group (*p* = 0.046). Additionally, the proportion of male patients and patients with non-smoking histories were significantly higher in the TDT ≥400 d group than in the TDT < 400 d group (both *p* < 0.001). Finally, the proportion of adenocarcinomas was significantly lower in the TDT < 400 d group than in the TDT ≥400 d group (*p* < 0.001).

### Relationships between TDT and OS and RFS in all patients

**Fig. 2:**
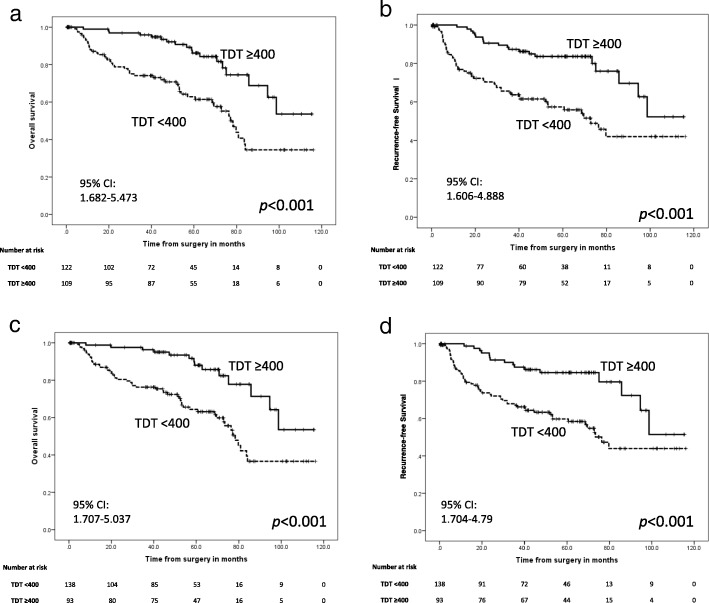
Kaplan–Meier OS and RFS curves for patients divided according to TDT for all components versus solid components. **a** OS of all components, **b** RFS of all components, **c** OS of solid components, and (**d**) RFS of solid components. TDT: Tumor Doubling Time, 95% CI: 95% Confidence Interval

Figure 2 shows OS and RFS curves for all patients according to TDT of all components and TDT of solid components. The all components TDT < 400 d group had significantly poorer OS (Fig. 2a) and RFS (Fig. 2b) (both *p* < 0.001). Similarly, the solid components TDT < 400 d group had significantly poorer OS (Fig. 2c) and RFS (Fig. 2d) (both *p* < 0.001).

### Clinical characteristics of pathological stages IA1, IA2, IA3 and IB divided by TDT

Next, we evaluated whether solid component TDT influenced pathological stage I cases (IA1, IA2, IA3 and IB), as the solid component has become more important for stage I patients in the 8th edition of the American Joint Committee for Cancer Staging System. Table 2 shows the clinical characteristics of patients divided into pathological stage IA1 (*n* = 49), IA2 (*n* = 61), IA3 (*n* = 20) and IB (*n* = 46). The mean age was significantly higher in the TDT < 400 d group than in the TDT ≥400 d group among Stage IA1 and IA3 patients (*p* = 0.02 and *p* = 0.03, respectively). Additionally, the proportion of adenocarcinomas was significantly lower in the TDT < 400 d group than in the TDT ≥400 d group among Stage IA2 patients; there were no other significant differences among other stages.

**Table 2:** Patient characteristics according to TDT (< 400 and ≥ 400) in pathological stages IA1, IA2, IA3 and IB (*n* = 176)

	Stage IA1 (*n* = 49)			Stage IA2 (*n* = 61)			Stage IA3 (*n* = 20)			Stage IB (*n* = 46)		
	TDT < 400	TDT ≥ 400		TDT < 400	TDT ≥ 400		TDT < 400	TDT ≥ 400		TDT < 400	TDT ≥ 400	
	n = 17	*n* = 32	*p-value*	*n* = 34	*n* = 27	*p-value*	*n* = 13	*n* = 7	*p-value*	*n* = 31	*n* = 15	*p-value*
Age (mean ± SD)	73.9 ± 7.8	68.1 ± 8.3	0.02	69.4 ± 10.9	66.5 ± 10.1	0.299	75.4 ± 5.2	63.3 ± 11.1	0.03	72.3 ± 7.6	72.5 ± 9.6	0.966
Sex (Male/Female)	6 (35.3%)/11 (64.7%)	11 (34.4%)/21 (65.6%)	0.949	26 (76.5%)/8 (23.5%)	13 (48.1%)/14 (51.9%)	0.022	10 (76.9%)/3 (23.1%)	4 (57.1%)/3 (42.9%)	0.357	24 (77.4%)/7 (22.6%)	11 (73.3%)/4 (26.7%)	0.761
Smoking History (Never/Ever)	5 (29.4%)/12 (70.6%)	8 (25%)/24 (75%)	0.739	22 (64.7%)/12 (35.3%)	11 (40.7%)/16 (59.3%)	0.062	10 (76.9%)/3 (23.1%)	4 (57.1%)/3 (42.9%)	0.357	26 (83.9%)/5 (16.1%)	10 (66.7%)/5 (33.3%)	0.257
Serum CEA (mean ± SD)	2.2 ± 1.1	2.8 ± 3.1	0.488	2.6 ± 1.8	3.2 ± 1.7	0.223	10.7 ± 24.4	2.8 ± 1.1	0.408	4.2 ± 3.0	9.3 ± 20.0	0.172
Type of resection												
Segmentectomy	6 (35.3%)	8 (25%)	0.448	5 (14.7%)	3 (11.1%)	0.68	0	0	–	0	1 (6.7%)	0.326
Lobectomy	11 (64.7%)	24 (75%)		29 (85.3%)	24 (88.9%)		13 (100%)	7 (100%)		31 (100%)	14 (93.3%)	
Pneumonectomy	0	0		0	0		0	0		0	0	
Histological type												
Adenocarcinoma	16 (94.1%)	31 (96.9%)	0.578	21 (61.8%)	25 (92.6%)	0.021	7 (53.8%)	6 (85.7%)	0.319	18 (58.1%)	10 (66.7%)	0.802
Squamous cell carcinoma	1 (5.9%)	0		7 (20.6%)	1 (3.7%)		4 (30.7%)	1 (14.3%)		9 (29%)	3 (20%)	
Others	0	1 (3.1%)		6 (17.6%)	1 (3.7%)		2 (15.4%)	0		4 (12.9%)	2 (13.3%)	
Solid component												
Pure solid/mixed GGO	10/7	11/22	0.084	19/15	11/16	0.24	6/7	2/5	0.44	10/21	5/10	0.94

*TDT* Tumor Doubling Time, *SD* Standard deviation

### Relationships between TDT and OS and RFS in stage I patients

**Fig. 3:**
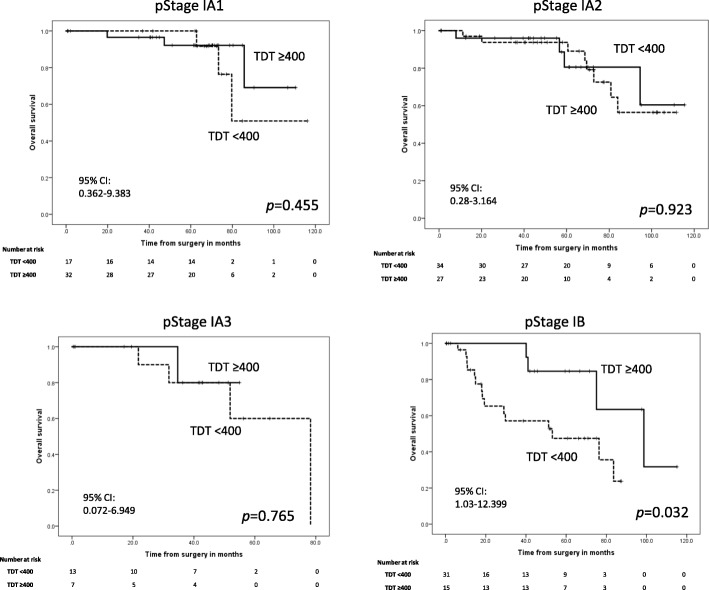
Kaplan–Meier OS curves for patients divided according to TDT and pathological stage. OS curves using the Kaplan–Meier method with patients divided by pathological stage as indicated. TDT: Tumor Doubling Time, 95% CI: 95% Confidence Interval

**Fig. 4:**
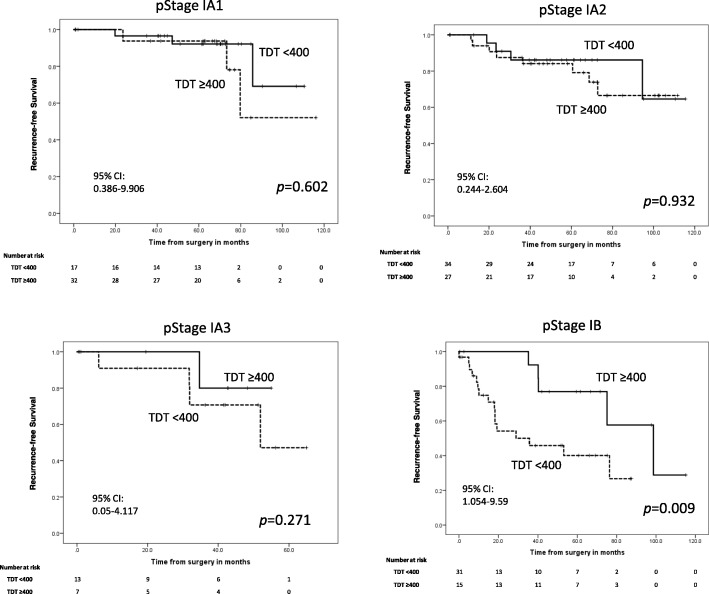
Kaplan–Meier RFS curves for patients divided according to TDT and pathological stage. RFS curves using the Kaplan–Meier method with patients divided by pathological stage as indicated. TDT: Tumor Doubling Time, 95% CI: 95% Confidence Interval

Figures 3 and 4 show the relationships between solid component TDT and OS and RFS in pathological stage IA1, IA2, IA3 and IB cases. The OS and RFS of the TDT < 400 d group were significantly reduced for Stage IB patients (*p* = 0.032 and *p* = 0.009, respectively). There were no significant differences among stage IA1, IA2 or IA3 patients for either OS or RFS after segregating cases based on TDTs.

### Examining whether solid component TDT or all component TDT affect prognosis

**Fig. 5:**
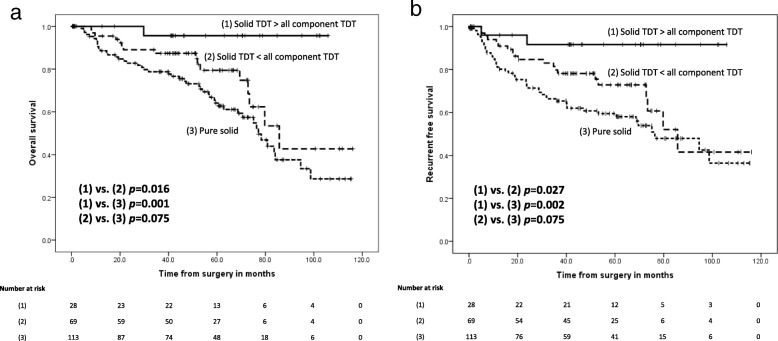
Kaplan–Meier OS and RFS curves for patients divided according to TDT differences between solid and whole tumor components. **a** OS and **b** RFS curves using the Kaplan–Meier method with patients divided into (1) those with a TDT of solid components > TDT of all components, (2) those with TDT of solid components < TDT of all components, and (3) those with TDT of solid components = TDT of all components, which meant pure solid tumors. TDT: Tumor Doubling Time, 95% CI: 95% Confidence Interval

Next, we evaluated whether all component TDT or solid component TDT had a greater effect on prognosis. To this end, we divided the patients into three groups: (1) patients whose solid component TDT was longer than their all component TDT, (2) patients whose solid component TDT was shorter than their all component TDT, and (3) patients whose solid component TDT and all component TDT were equal, i.e. pure solid tumors. Patients with infinite TDTs were excluded from this sub-analysis, and there were no patients with pure GGO tumors in this study. Figure 5 shows Kaplan–Meier curves for each group. The group with the worst prognosis (OS and RFS) from this analysis was group 3, the pure solid tumor group. Additionally, we found that group 2 had significantly poorer prognoses than group 1 for both OS and RFS (*p* = 0.016 and *p* = 0.027, respectively). Thus, this analysis demonstrated that patients with faster growing solid components have poorer outcomes than patients that have growth throughout the GGO components of the tumor.

### Prognostic factors in each group

Univariate analysis among all patients showed that sex, pathological stage, smoking history, histological type and solid component TDT were significant predictors of OS. Cox hazard model showed that pathological stage and shorter solid component TDT were independent risk factors of reduced OS (*p* = 0.001 and *p* = 0.022, respectively). Additionally, sex, pathological stage, smoking history, histological type and solid component TDT were significant predictors of RFS in univariate analysis, and sex, pathological stage and solid component TDT were independent risk factors for reduced RFS in multivariate analysis (*p* = 0.013, *p* < 0.001 and *p* = 0.033, respectively) (Table 3).

**Table 3:** Univariate and multivariate Cox proportional hazard regression analysis of the clinicopathological parameters of all patients (*n* = 231)

	Univariate			Multivariate		
	HR	95%CI	*p*-value	HR	95%CI	*p*-value
Overall survival						
Age (< 70)	0.628	0.366–1.077	0.091			
Sex (Female)	0.49	0.368–0.651	< 0.001	0.459	0.177–1.192	0.11
pStage (II–IV)	3.291	2.061–5.235	< 0.001	2.344	1.4–3.924	0.001
Smoking history (Yes)	3.85	2.257–6.568	< 0.001	1.346	0.533–3.403	0.53
Histological type (non-adeno)	3.272	2.078–5.153	< 0.001	1.327	0.776–2.271	0.302
Solid TDT (< 400 d)	3.034	1.682–5.473	< 0.001	2.093	1.111–3.944	0.022
Recurrence-free survival						
Age (< 70)	1.331	0.786–2.254	0.287			
Sex (Female)	0.639	0.494–0.827	0.001	0.318	0.128–0.788	0.013
pStage (II–IV)	4.067	2.537–6.52	0.001	2.846	1.662–4.874	< 0.001
Smoking history (Yes)	2.161	1.325–3.525	0.002	0.489	0.197–1.211	0.122
Histological type (non-adeno)	2.897	1.811–4.633	< 0.001	1.617	0.894–2.922	0.112
Solid TDT (< 400 d)	3.152	1.673–5.939	< 0.001	2.099	1.063–4.144	0.033

*HR* Hazrad Ratio, *95% CI* 95% Confidence Interval

## Discussion

This study highlighted some important clinical aspects of NSCLC. First, NSCLC patients with shorter solid component TDTs had significantly poorer prognoses than patients with longer TDTs, which indicate indolent disease; additionally, we found that the solid component TDT was more predicative of outcomes than all component TDT. Second, among pathological stage I patients, shorter solid component TDTs only significantly influenced prognosis in stage IB patients, but not stage IA1, IA2 or IA3 patients, although the patient numbers for this analysis were low. Additionally, patients with shorter solid component TDTs had significantly poorer prognoses than patients with shorter all component TDTs.

In this study, solid component TDT < 400 d was an independent prognostic risk factor for patients with resected NSCLC. Previous reports have suggested that TDT < 400 d is the best cut-off value to distinguish indolent versus malignant lesions, and that TDT could be a key parameter for distinguishing aggressive from slow-growing cancers [4, 5, 8]. In this study, the proportion of adenocarcinomas was higher in the TDT ≥400 d group than in the TDT < 400 d group. We suggest that this finding is related to tumor-specific properties. Previous reports investigating TDT in lung cancer using chest CT scans have found that the TDTs of adenocarcinomas are longer than squamous cell carcinomas [11–14]. However, well-differentiated adenocarcinomas, which are known to be slow growing, may have affected these results.

In the sub-analysis of OS and RFS limited to Stage I patients, solid component TDT was found to only influence stage IB patients. Recently, the solid component of lung cancer in chest CT scans was indicated to be more important for tumor classification by the American Joint Committee for Cancer Staging System 8th edition, as it has been shown to affect prognosis more than GGO components. Based on our results, we suggest that pathological stage IB patients should specifically be re-imaged by chest CT scan to calculate their solid component TDT after surgical resection. Then, if their solid component TDT is < 400 d, more aggressive postoperative adjuvant therapy and more frequent follow-ups should be given.

The OS and RFS curves for all patients sorted by all component TDT and solid component TDT indicated that patients with short TDTs (< 400 d) in either solid or all components had significantly poorer prognoses than patients with long TDTs (≥400 d). Indeed, the relationship between solid component TDT and NSCLC prognosis were almost equal to the relationship between all component TDT and NSCLC prognosis for this cohort. Thus, we further evaluated whether solid component TDT or all component TDT were better associated with the prognosis of completely resected NSCLC patients. Our data showed that pure solid tumors, which were defined as having the same TDT for solid components and all components, had a poorer prognosis than mixed GGO tumors. Thus, in patients with mixed GGO tumors, the solid component TDT was more correlative to prognosis than the all component TDT. This result suggested that the solid component TDT should be emphasized more than the all component TDT when pre- or post-operative evaluation chest CT scans are evaluated.

Other studies have investigated TDT in different solid cancers with imaging modalities [15–20], and generally, they have concluded that shorter TDTs are correlated with tumor malignancy and poor prognoses. For example, Choe et al. reported that there was a negative relationship between TDT (shorter doubling times) and aggressive histological phenotypes in thymic epithelial tumors [18]. Kim et al. reported that faster hepatocellular carcinoma growth rates were associated with reduced liver function and survival and increased recurrence rates [16]. Ryu et al. showed that TDTs differed significantly among three molecular breast cancer subtypes (ER+, HER2+ and triple-negative) using ultrasound. Regarding NSCLC, although Nakamura et al. have shown the relationship between EGFR mutation status and TDT, additional studies on relationships between different lung cancer subtypes and TDT are needed.

There were several limitations to this study. First, patients who did not undergo two thin-slice CT scans before surgery were excluded (*n* = 138 patients). Additionally, patients who could not be measured accurately because of inflammatory changes or obstructive pneumonia surrounding the tumor or lung cavity were excluded (*n* = 151). These unmeasurable patients tended to have advanced-stage disease, and their exclusion could have affected the results. Thus, there is the possibility of selection bias being introduced. Second, the patient cohort was relatively small, particularly when we needed to subdivide the cases into four pathological stage I sub-groups. We would like to further accumulate cases and reconsider them in future studies. And then, the radiological evaluations have been performed by two thoracic surgeons. Although it performed under the guidance of radiologist, there might be a slight measurement error.

## Conclusions

In conclusion, resected NSCLC patients with short solid component TDTs (< 400 d) had significantly poorer prognoses than patients with long either all or solid component TDTs (≥400 d). When restricted to pathological stage I patients, short solid component TDT was only significant for stage IB patients, but not stage IA1, IA2 or IA3 patients, although patient numbers were quite low. Additionally, patients with short solid component TDTs had significantly poorer prognoses than patients with short all component TDTs. Thus, clinicians should pay special attention to the doubling time of the solid component during follow-up observation.

## Abbreviations

CEA: Carcinobryonic antigen
CT: Computed tomography
GGO: Grand-glass opastiy
MRI: Magnetic resonance imaging
NSCLC: Non-small cell lung cancer
OS: Overall survival, RFS; Recurrent free survival
TDT: Tumor doubling time
